# Revalidating the prognostic COVID-19 severity assessment (COSA) score for variants of concern

**DOI:** 10.1186/s12967-022-03634-x

**Published:** 2022-09-23

**Authors:** Verena Schöning, Evangelia Liakoni, Christine Baumgartner, Aristomenis K. Exadaktylos, Wolf E. Hautz, Andrew Atkinson, Felix Hammann

**Affiliations:** 1 Clinical Pharmacology and Toxicology, Department of General Internal Medicine, Inselspital, Bern University Hospital, University of Bern, Bern, Switzerland; 2 Department of General Internal Medicine, Inselspital, Bern University Hospital, University of Bern, Bern, Switzerland; 3 Department of Emergency Medicine, Inselspital, Bern University Hospital, University of Bern, Bern, Switzerland; 4 Pediatric Pharmacology and Pharmacometrics Research Group, University Children’s Hospital, Basel, Switzerland; 5 Department of Infectious Diseases, Inselspital, Bern University Hospital, University of Bern, Bern, Switzerland

Dear Editor,

Many prediction models have been published for Coronavirus disease 2019 (COVID-19) to support clinical decision-making for either diagnosis, prediction of mortality risk, or disease progression. An ongoing review concluded that most of these were poorly reported and had a high risk of bias, casting doubt over their real-world predictive value [1]. Additionally, dominant strains are no longer the ancestral type but variants of concern (VOCs) with different pathogenicity, and vaccinations and herd immunity can influence individual outcomes. We aimed to assess the performance of our previously published COSA (COVID-19 Severity Assessment) score on admitted patients in the twelve months following its development, a time during which the score was used clinically for risk stratification.

The COSA score was developed using data from patients who tested positive for severe acute respiratory syndrome coronavirus 2 (SARS-CoV-2) by reverse-transcriptase polymerase chain reaction (RT-PCR) between February 1st and November 16th, 2020 (1st and 2nd waves in Switzerland, i.e. the original cohort) [2]. The revalidation cohort consisted of patients who tested positive for SARS-CoV-2 between November 17th, 2020 and November 16th, 2021. The primary outcome was disease severity, as determined by the worst outcome at any point after diagnosis:

- Non-severe outcome: no intensive care unit (ICU) admission or death of any cause during the observational period
- Severe outcome: ICU admission at any stage and / or death of any cause

All patients were discharged or had died by the time the revalidation was performed.

The COSA score (Table 1) was calculated for each patient using the most extreme laboratory values within 3 days prior to 1 day after the positive SARS-CoV-2 test. The results of the original and the revalidation cohort were then compared.

**Table 1:** COVID-19 severity assessment (COSA) score (from Ref. [2])

Parameter	Value	Score points
Sex	Male	1
CRP	≥ 25 mg/L	3
Sodium	≥ 144 mmol/L	2
Hemoglobin	≤ 100 g/L	1
eGFR according to CKD-EPI	≤ 75 mL/min	1
Glucose	≥ 8.6 mmol/L	1
Leucocytes	≥ 10 G/L	1

The COSA score was calculated for each patient using the most extreme values within 3 days prior to 1 day after the positive SARS-CoV-2 test, with a score of 6 or higher indicating a high risk (> 50%) for a severe progression

Data wrangling, analysis, and visualization were performed in GNU R (version 4.0.2, [3]). Statistical significance levels were defined at a p-value of < 0.05, and determined with the Wilcoxon rank sum test for continuous variables, and the Chi-square test for categorical variables using the *stats* package (version 4.0.2). The area under the receiver operating characteristic curve (AUROC) and the 95% confidence intervals (CI) were calculated to assess the discriminatory power of the COSA score to predict a severe outcome. The COSA score was considered validated if the 95% CI of the AUROC from fitting the original and the revalidation data set were overlapping.

The original cohort consisted of 626 patients (457 non-severe and 169 severe outcomes), and 508 patients were included in the revalidation cohort (301 non-severe and 208 severe outcomes). A comparison of the demographics and laboratory parameters of both cohorts is provided in Table 2. The patients in the original non-severe cohort were significantly younger with a lower share of inpatients than in the non-severe revalidation cohort. The latter had significantly higher peak C-reactive protein (CRP) and glucose, and lower minimal hemoglobin and estimated glomerular filtration rate values. For severe patients, we noted a higher proportion of inpatients and deaths, and significantly lower body weights and body mass index in the revalidation cohort. There was no significant difference in laboratory parameters.

**Table 2:** General demographics and laboratory parameters of the original and revalidation cohort

	Non-severe (N = 758)			Severe (N = 512)		
	Original (N = 457)	Revalidation (N = 301)	P value	Original (N = 171)	Revalidation (N = 208)	P value
Demographics						
Age (years)						
Median (IQR)	64.00 (49.00, 76.00)	72.00 (59.00, 82.00)	** < 0.002**	68.00 (57.00, 78.00)	72.00 (59.75, 82.25)	0.058
Sex						
Female, n (%)	188 (41.05)	125 (41.81)	0.895	45 (26.32)	63 (30.29)	0.460
Hospitalization						
Inpatients, n (%)	319 (69.58)	299 (100.00)	** < 0.002**	163 (95.27)	208 (100.00)	**0.005**
Deaths						
Deceased, n (%)	0 (0.00)	0 (0.00)	–	51 (29.82)	120 (57.69)	** < 0.002**
Weight (kg)						
Median (IQR)	77.90 (66.23, 88.00)	76.30 (65.62, 88.00)	0.526	81.00 (70.40, 93.70)	75.20 (65.20, 90.00)	**0.026**
Height (cm)						
Median (IQR)	170.00 (165.00, 176.00)	170.00 (163.00, 177.00)	0.241	170.00 (165.00, 176.00)	170.00 (165.00, 176.75)	0.883
Body Mass Index (BMI, kg/m2)						
Median (IQR)	25.98 (23.38, 29.74)	26.20 (23.29, 30.32)	0.489	28.07 (25.20, 31.20)	26.87 (23.28, 29.81)	**0.018**
Laboratory parameters						
Maximum C-reactive protein (CRP) levels						
Median (IQR)	37.00 (10.00, 78.00)	60.00 (21.00, 107.50)	** < 0.002**	106.00 (59.00, 175.00)	125.50 (62.00, 202.50)	0.300
Maximum sodium levels						
Median (IQR)	139.00 (137.00, 141.00)	139.00 (137.00, 142.00)	0.262	142.00 (139.00, 145.00)	142.00 (138.00, 146.00)	0.901
Minimum hemoglobin levels						
Median (IQR)	132.00 (118.00, 144.00)	126.00 (111.00, 142.00)	**0.004**	113.00 (89.50, 125.00)	107.50 (84.75, 124.25)	0.372
Minimum glomerular filtration rate (GFR) values						
Median (IQR)	82.00 (61.00, 97.00)	75.00 (52.00, 93.00)	**0.007**	64.00 (39.50, 87.00)	56.00 (31.75, 84.25)	0.158
Minimum glucose values						
Median (IQR)	6.40 (5.67, 7.90)	6.90 (6.00, 8.46)	**0.002**	9.50 (7.46, 12.55)	9.45 (7.18, 11.80)	0.393
Minimum leukocytes values						
Median (IQR)	6.32 (4.70, 8.61)	6.41 (4.91, 9.09)	0.235	8.92 (6.65, 13.70)	10.90 (7.03, 15.12)	0.072

Laboratory parameters were considered from three day prior to until 1 day after the first positive SARS-CoV-2 PCR test result

*IQR* interquartile range

Bold numbers indicate significant differences (p < 0.05) between severe and non-severe cases.

The AUROC was 0.85 (95% CI 0.82–0.88) and 0.80 (95% CI 0.76–0.84) for the original and revalidation cohort, respectively (Fig. 1). A closer look at the severity distribution per score value (Fig. 2) revealed a greater share of severe cases with low score values (0–4 points) in the revalidation cohort than in the original cohort. No major differences are noticeable for score points greater than 4.

**Fig. 1:**
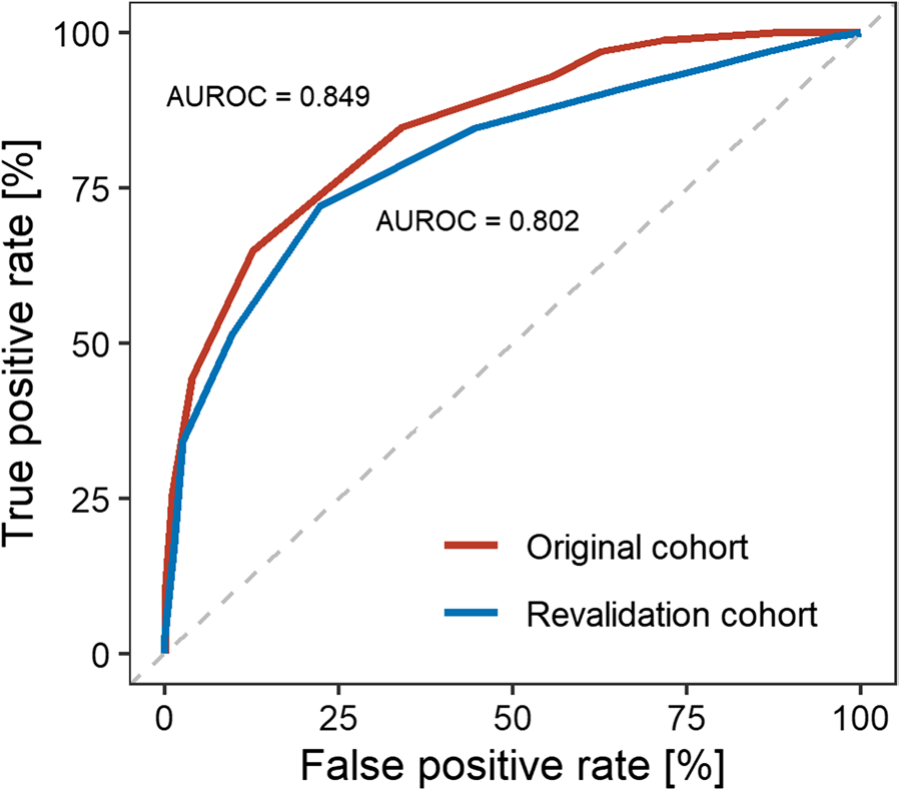
Area under the receiver operating characteristic (AUROC) of the COVID-19 severity assessment (COSA) score. Original (red) and revalidation cohort (blue)

**Fig. 2:**
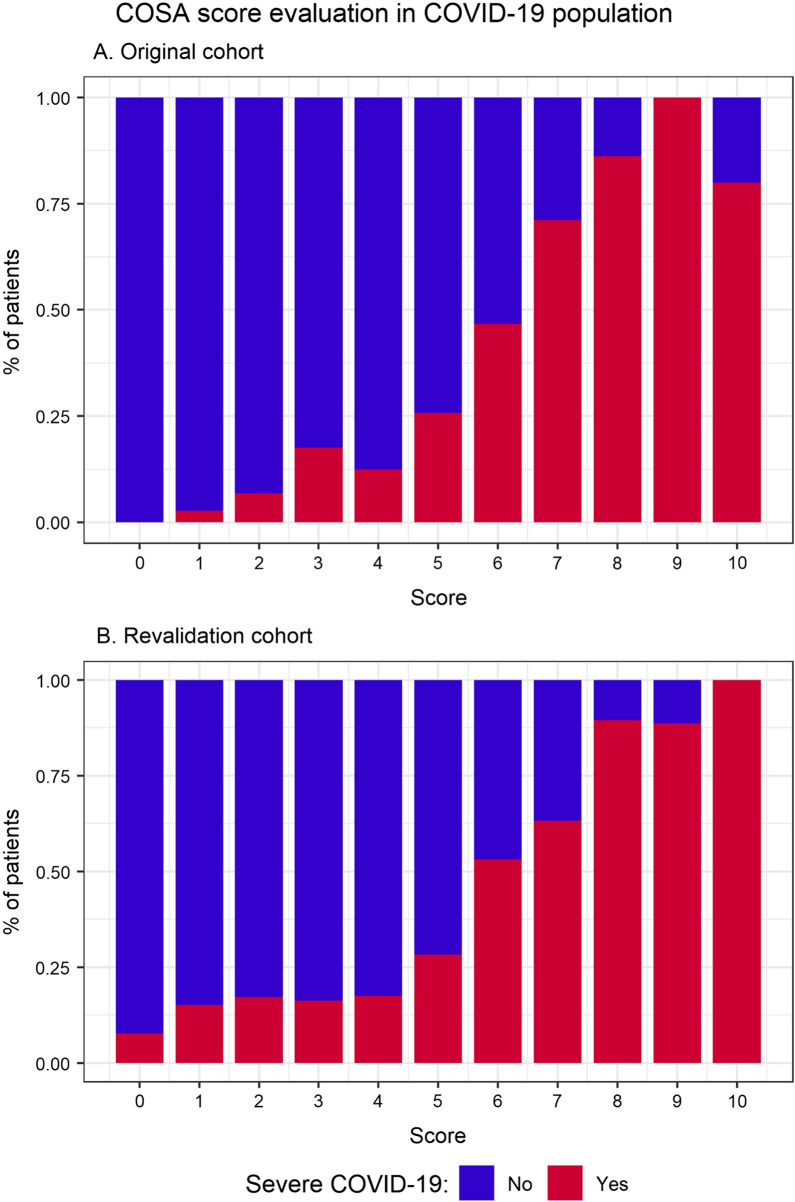
Percentage amount of patients with severe and non-severe COVID-19 in relation to score points in the original (**A**) and revalidation (**B**) cohort

Patients in the revalidation cohort presented with overall worse laboratory markers and a greater likelihood of severe outcomes compared to the original cohort (69.1% vs. 37.4%). This could be due to more selective laboratory testing for hospitalized patients only and differences in circulating variants. Although no sequencing data is available for either cohort, national surveys indicate that the dominant strain during the 1st and 2nd waves (original cohort) was the ancestral type, whereas the 3rd and 4th waves (revalidation cohort) were driven by the more transmissible and virulent VOCs Alpha (B.1.1.7) and Delta (B.1.617.2) [4]. Vaccination campaigns and an emerging population-level immunity likely mitigated the individual disease severity [5].

While the performance of the score remains robust with strong positive discriminative ability we did note a decrease in specificity towards the lower end of the scale (Fig. 2). This suggests that readjustment of the cut-offs might be beneficial for a better separation of the severity classes as new variants emerge.

Despite changes in the viral landscape and population immunity, the COSA score still delivered good predictions of disease progression one year after its development. We attribute this to the simple set of covariates and the rigorous internal and external validation in the original model-building process. Adaptations to the score could become necessary in the near future as vaccination effects begin to wane and if drastically different VOCs appear.

## Data Availability

The source code and corresponding input file are available on GitHub: https://github.com/cptbern/COSAscore. The datasets used and/or analysed during the current study are available from the corresponding author on reasonable request.

## Abbreviations

AUROC: Area under the receiver operating characteristic
BMI: Body mass index
COSA: COVID-19 severity assessment
COVID-19: Coronavirus disease 19
CRP: C-reactive protein
eGFR: Estimated glomerular filtration rate
ICU: Intensive Care Unit
RT-PCR: Reverse-transcriptase polymerase chain reaction
SARS-CoV-2: Severe acute respiratory syndrome coronavirus 2
VOC: Variants of concern
